# Similar Short-Term Outcomes of Adolescent Idiopathic Scoliosis Surgery with or without Drainage: A Systematic Review of the Literature and Meta-Analysis

**DOI:** 10.3390/jpm14040339

**Published:** 2024-03-24

**Authors:** Alberto Ruffilli, Matteo Traversari, Giovanni Viroli, Marco Manzetti, Marco Ialuna, Manuele Morandi Guaitoli, Antonio Mazzotti, Elena Artioli, Simone Ottavio Zielli, Alberto Arceri, Cesare Faldini

**Affiliations:** 1 IRCCS Istituto Ortopedico Rizzoli, 1st Orthopaedics and Traumatology Clinic, University of Bologna, 40136 Bologna, Italy; alberto.ruffilli@ior.it (A.R.); matteo.traversari@ior.it (M.T.); giovanni.viroli@ior.it (G.V.); marco.manzetti@ior.it (M.M.); marco.ialuna@ior.it (M.I.); manuele.morandiguaitoli@ior.it (M.M.G.); simoneottavio.zielli@ior.it (S.O.Z.); alberto.arceri@ior.it (A.A.);; 2 Department of Biomedical and Neuromotor Sciences, University of Bologna, 40123 Bologna, Italy

**Keywords:** adolescent idiopathic scoliosis, scoliosis surgery, drainage, drain, transfusion

## Abstract

The use of closed suction drains post posterior spinal fusion for adolescent idiopathic scoliosis (AIS) is common practice, although evidence on its impact is limited compared to that for knee and hip arthroplasty. This study aimed to assess the effect of closed suction drainage on short-term post-operative outcomes in AIS surgery. A systematic review following PRISMA guidelines was conducted, including studies comparing outcomes with and without drainage. Data on blood loss, transfusions, hospital stay, and complications were collected and subjected to meta-analysis. Five studies involving 772 patients were analyzed. The meta-analysis found no significant difference in blood transfusion rates (*p* = 0.107) or hospital stay (*p* = 0.457) between groups. Complications, including surgical site infections, were more common without drainage, though not statistically significant (*p* = 0.356). Reintervention rates were higher in the no-drainage group, but not significantly (*p* = 0.260). Overall, this review found no significant short-term outcome differences, suggesting clinical judgment should guide drainage decisions. Further research, particularly with enhanced recovery protocols, is warranted to clarify drainage’s role in AIS surgery.

## 1. Introduction

Surgical treatment of adolescent idiopathic scoliosis is indicated in patients affected by severe or progressive spinal curvature [1]. However, it is important to note that these patients are generally healthy individuals with moderate-to-severe spinal deformities, whose treatment has a mainly aesthetic purpose, since cardiovascular and respiratory systems disruption starts to be a concern only with severe deformities in terms of Cobb angle [2]. Therefore, the peri-operative and post-operative course should be as fast and free of complications as possible to favor the benefits over the risks. Closed suction drain placement is, in most cases, a well-established practice that is routinely implemented following posterior spinal fusion for AIS [3], but, differently to what happens in total knee and hip arthroplasty, there is a substantial lack of evidence regarding a positive, neutral, or negative effect of drainage on short-term post-operative outcomes [4]. There is a paucity of quality literature examining whether drains could have an impact on infection rate, complication rate, blood transfusion rate, and length of hospital stay. The aim of the present work is to systematically review the available literature regarding the effect of closed suction drainage in short-term post-operative outcomes of AIS surgery.

## 2. Materials and Methods

A systematic review of the literature regarding the use of closed suction drainage in adolescent idiopathic scoliosis surgery was conducted in accordance with the PRISMA (preferred reporting items for systematic reviews and meta-analyses) guidelines [5].

Only peer-reviewed publications were considered for inclusion. Studies which compared the outcomes of patients who underwent surgical correction of AIS through the posterior-only approach with and without the use of closed suction drainage were included. Only articles in English that met the PICO (Population, Intervention, Comparison, and Outcomes) criteria on systematic reviews were included.

Only randomized controlled trials (RCTs), prospective and retrospective cohort studies (PCS and RCS) were considered for inclusion. In vitro studies and animal model studies were excluded, as well as case reports and case series.

This systematic review has not been registered.

### 2.1. Search Strategy

Studies eligible for this systematic review were identified through an electronic systematic search of PubMed and the Cochrane Central Registry of Controlled Trials until August 2023.

The following search strings were used:

- Pub-Med: “(Drain OR drainage) AND (scoliosis OR adolescent idiopathic scoliosis OR AIS)”
- Cochrane library: “drainage AND scoliosis”.

### 2.2. Study Selection

**Figure 1:**
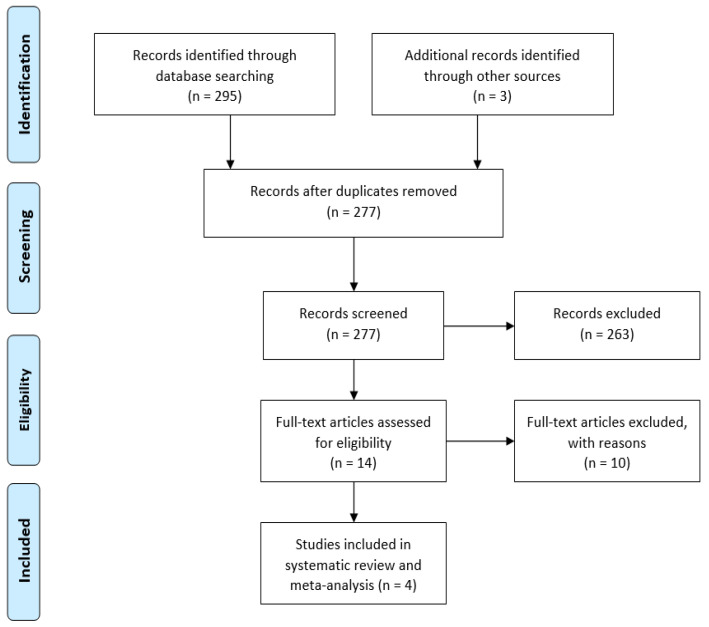
PRISMA flowchart showing the study selection process.

Articles considered relevant by electronic search were retrieved in full-text, and hand searches of their bibliographies were performed to find further related articles. Reviews and meta-analyses were also analyzed to identify potentially missed eligible papers. Duplicates were removed. The study selection process was carried out in accordance with the PRISMA flowchart (Figure 1). After full text screening, records that did not meet the eligibility criteria were excluded. Remnant studies were categorized by type, according to the Oxford Centre for Evidence-Based Medicine (OCEBM).

The quality of the included studies was evaluated using the Risk of Bias in Non-randomized Studies of Interventions (ROBINS-I) tool [6] if they were retrospective comparative studies and Revised Cochrane risk-of-bias tool for randomized trials (RoB 2) [7] if they were clinical trials.

### 2.3. Data Collection Process

All included studies were analyzed, and data related to post-operative outcomes of interest were extracted and summarized by two distinct authors (M.T. and M.M.).

Meta-analyses were performed when there were at least three studies to be compared. Heterogeneity between studies was assessed using the inconsistency statistic (I2 > 75% was considered as high heterogeneity). Publication biases were assessed with Egger’s test and represented with forest plots. Log odds ratios or standardized mean differences were used as measures of effect size. A random effect model was applied. A *p*-value of <0.05 was considered to be significant. All statistical analyses were conducted with Jamovi version 2.2 (The Jamovi Project, Sydney, Australia) software.

## 3. Results

### 3.1. Baseline Studies Characteristics and Quality Assessment

**Figure 2:**
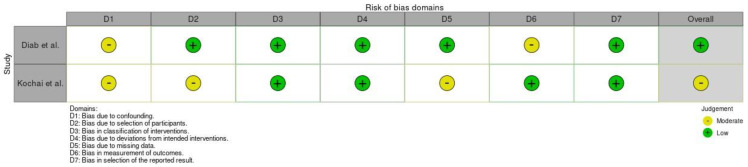
Quality assessment process of studies using ROBINS-I [3,11].

**Figure 3:**
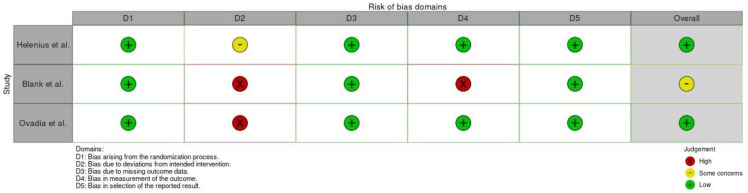
Quality assessment process of studies using RoB 2 [8,9,10].

A total of 298 studies were found through electronic search; after screening, five studies (three randomized clinical trials [8,9,10] and two retrospective comparative studies [3,11]) were included. Meta-analysis was conducted on randomized controlled trials and comparative studies. The quality of the papers was good in 3 cases and fair in 2 cases (Figure 2 and Figure 3).

Included studies are heterogeneous regarding the type of drainage used; drainage details are summarized in Table 1.

Three studies used subfascial closed suction drainage [8,10,11], Blank et al. [9] used subcutaneous closed suction drainage, and Diab et al. [3] reported the data of several surgeons who used different drains. Helenius et al., Ovadia et al. and Blank et al. pre-operatively randomized patients into either drain or no-drain groups; blindness of patients and surgeons was not possible [8,9,10]. In retrospective studies, the criteria for drainage placement were not completely clarified. Diab et al. [3] reported that drainage was used twice as often as not and there was a general tendency of spine surgeons to use drainages based on personal experience and intra-operative patients’ characteristics. Kochai et al. [11] did not report criteria for drainage placement.

Included studies differ regarding the drain removal criteria. Helenius et al. and Blank et al. employed time criteria, removing drainage 24 and 48 h after surgery, respectively [9,10]. Conversely, Ovadia et al. and Kochai et al. removed drainage when the daily output volumes were less than 100 and 50 mL, respectively [8,11]. Diab et al. [3] reported the data of several surgeons with different drain removal criteria; the average removal time was 57.2 ± 25.8 h after surgery.

### 3.2. Included Patients’ Characteristics

A total of 772 patients were included. The mean age at surgery ranged from 14.4 to 15.8 years. Lenke type [1] was reported on 742 patients: 355 Lenke 1 (47.8%), 165 Lenke 2 (22.2%), 70 Lenke 3 (9.4%), 26 Lenke 4 (3.5%), 73 Lenke 5 (9.8%), and 53 Lenke 6 (7.1%). As for constructs, Helenius et al. [10] and Kochai et al. [11] used all-pedicle screw construct, while Diab et al. reported the data regarding several institutions in which the most common instrumentation was hybrid constructs, followed by all-pedicle screw. Patients’ age at surgery ranged between 13.3 [9] to 15.8 [8]. Details regarding surgical treatment are summarized in Table 2.

The pre-operative Cobb angle of major curve varied from 55.0 [10] to 65.4 [8] degrees. The post-operative Cobb angle of major curve was reported only in the paper by Helenius et al. with a mean value of 17.0 degrees [10]. Mean fused levels ranged between 7.6 [9] to 11.7 [3].

Blank et al. [9] reported the routine use of iliac crest bone harvest used as autograft and the subsequent placement of a subfascial drain at the donor site; conversely, Ovadia et al. [8] reported the absence of bone harvest both from the iliac crest and spinous processes.

Regarding accessory procedures, Helenius et al. performed posterior column osteotomies (PCOs) in approximately 15% of their patients [10], while in the cohort reported by Diab et al., thoracoplasty was performed in 6–10% of patients [3].

### 3.3. Blood Loss and Transfusion

The majority of included studies reported total blood loss, which was estimated as a combination of intra-operative blood loss and post-operative drainage output volume. An intraoperative cell saver was used in the majority of the included studies [3,8,9].

Estimates of intra-operative blood loss varied, ranging from 565.0 mL [10] to 1091.6 mL [9]. Different intra-operative blood loss estimation systems were employed by different authors. Helenius et al. used the formula 70 mL/kg multiplied by the patient’s weight in kilograms, and wound dressings were weighed, excluding any used saline [10]. Blank et al. [9] utilized the volume of blood and hematocrit collected by the intra-operative cell saver system to estimate blood loss; subsequently, the estimation was corrected with weighted surgical sponges. Diab et al. [3] reported the data of several surgeons without mentioning intra-operative blood loss estimation methods. Kochai et al. and Ovadia et al. did not report intra-operative blood loss [8,11]. Notably, there was a slight decreasing trend in intra-operative estimated blood loss over time, with Helenius et al. [10], the most recent paper, reporting the lowest amount of intra-operative blood loss. Furthermore, intra-operative blood loss appeared similar across all reported cohorts, whether patients were in the drain group or the no-drain group.

Blood transfusion rates in the included patients varied widely, Diab et al. reported a blood transfusion rate of 80% [3], while in the cohort by Kochai et al. [11], only two patients underwent blood transfusion post-operatively. The overall transfusion rates in patients in the drain and no-drain groups were 62.8% (292/465) and 56.7% (174/307), respectively [11]. Only three studies reported complete data on postoperative transfusion rates [3,9,10].

Diab et al. [3] reported that drained patients were less likely to receive intraoperative blood transfusions than those without a drain (*p* = 0.003). On the contrary, more drained patients received postoperative transfusions than those without a drain (*p* < 0.001). But it is important to mention that there was no difference between the patients of the two cohorts when considering overall transfusion rate (*p* = 0.08). Helenius et al., Diab et al. and Blank et al. reported a higher overall transfusion rate in patients without drains compared with patients in which drainage was applied.

One hundred and forty-three patients of the drain group required post-operative transfusions (143/389, 36.8%), while 47 patients required post-operative transfusions in the no-drain group (47/231, 20.3%).

**Figure 4:**
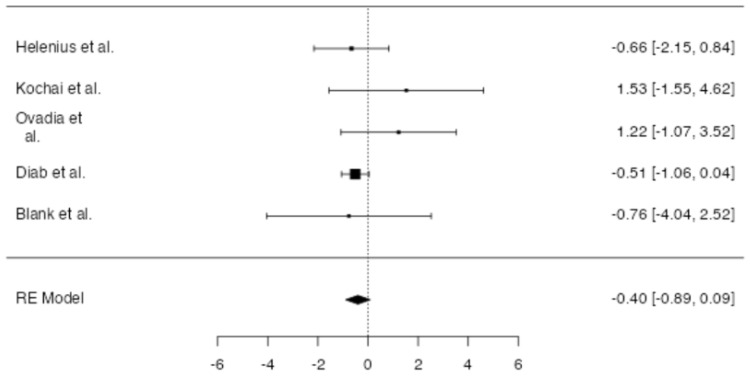
Forest plot of overall meta-analysis of the included studies with data about overall blood transfusion rate [3,8,9,10,11].

**Figure 5:**
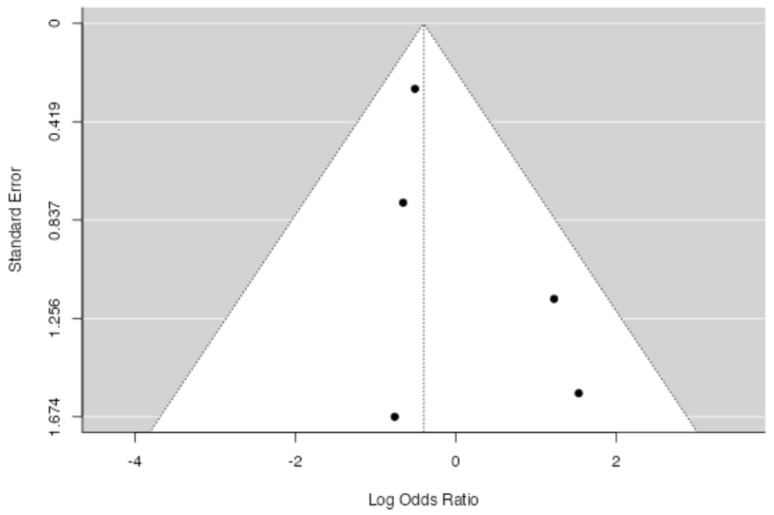
Funnel plot for assessing the presence of publication bias in the studies included in the meta-analysis regarding the overall transfusion rate.

No significant difference in overall blood transfusion rate between patients in which drainage was applied and patients without a drain was found at meta-analysis (*p* = 0.107, Figure 4), with an estimated average log odds ratio of −0.40 (95% CI −0.890–0.087), with a slight tendency of patients in the drain group to be less transfused. No significative heterogeneity (I2 = 0.0, *p* = 0.443) or publication bias (*p* = 0.232, Figure 5) were found.

### 3.4. Hospital Length of Stay

**Figure 6:**
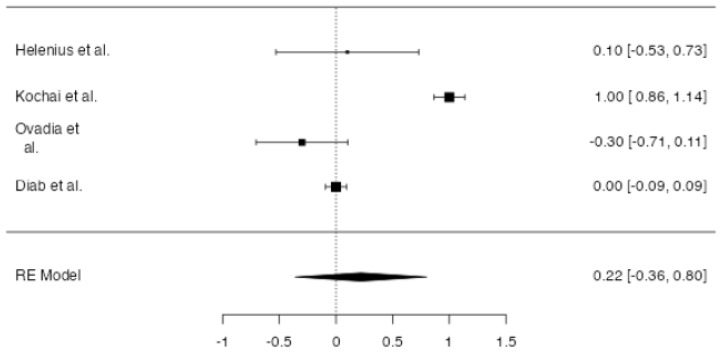
Forest plot of overall meta-analysis of the included studies with data about the length of hospital stay [3,8,10,11].

**Figure 7:**
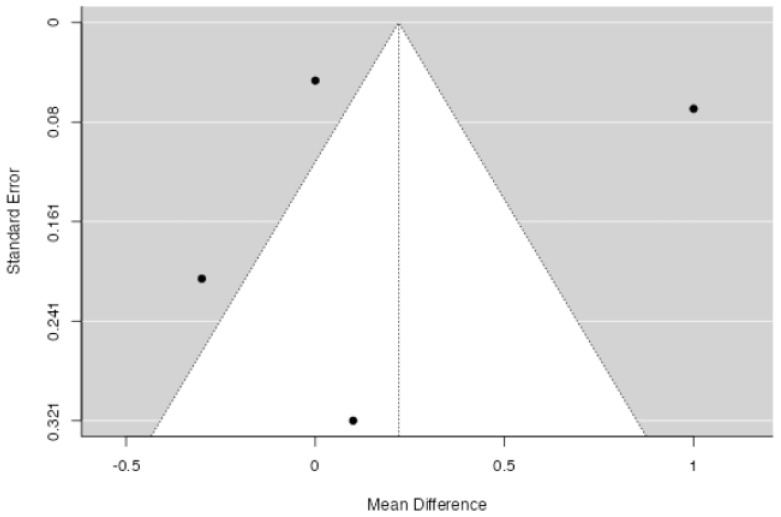
Funnel plot for assessing the presence of publication bias in the studies included in the meta-analysis regarding the length of hospital stay.

The length of stay of the included patients varied between 4.7 [10] and 6.1 [11] days. No significant difference in hospital length of stay between patients in which drainage was applied and patients without a drain was found at meta-analysis (*p* = 0.457, Figure 6), with an estimated average mean difference of +0.22 days for patients in the drain group (95% CI: −0.360 to 0.801 days). High heterogeneity (I2 = 97.25%, *p* < 0.001) was found. No publication bias (*p* = 0.523, Figure 7) was found.

### 3.5. Complications

The overall complication rates in patients in the drain and no-drain groups were 5.6% (26/465) and 10.7% (33/307), respectively. All authors reported a higher complication rate in the no-drain group, except for Kochai et al., who reported similar complication rates between the two groups [11]. Surgical site infection, both superficial and deep, was the most common post-operative reported complication, with an incidence ranging between 0% [8] and 17.8% [11] and between 0% [9] and 16.6% [11], for the drain and no-drain groups, respectively.

**Figure 8:**
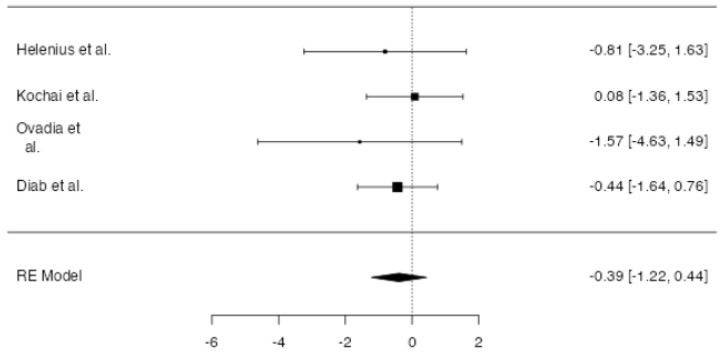
Forest plot of overall meta-analysis of the included studies with data about the surgical site infections [3,8,10,11].

**Figure 9:**
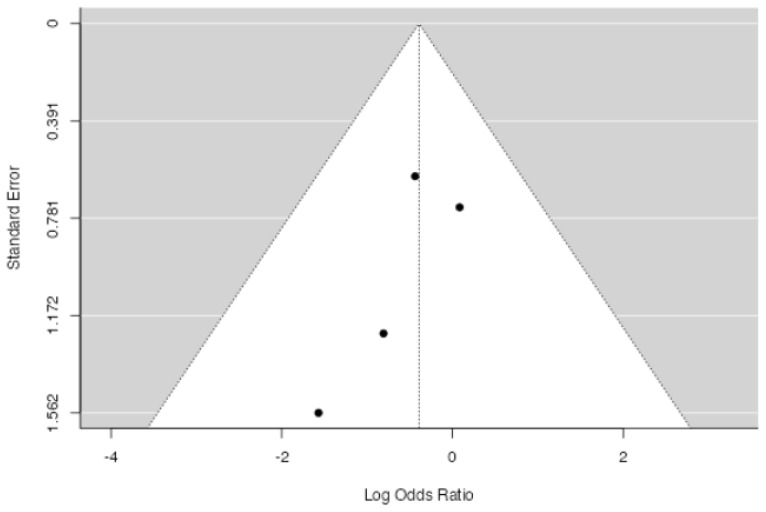
Funnel plot for assessing the presence of publication bias in the studies included in the meta-analysis regarding surgical site infections.

No significant difference in the postoperative surgical site infection rate between patients in which drainage was applied and patients without a drain was found at meta-analysis (*p* = 0.356, Figure 8), with an estimated average log odds ratio of −0.39 (95% CI −1.223–0.439). No significative heterogeneity (I2 = 0.0, *p* = 0.777) or publication bias (*p* = 0.447, Figure 9) were found.

Regarding non-infectious wound-related complications, patients in the no-drain group showed a higher overall complication rate, with a total of five complications (1.1%, wound dehiscence, large hematomas or sterile sieroma) compared to only one case (0.3%) of wound dehiscence in the drainage group.

**Figure 10:**
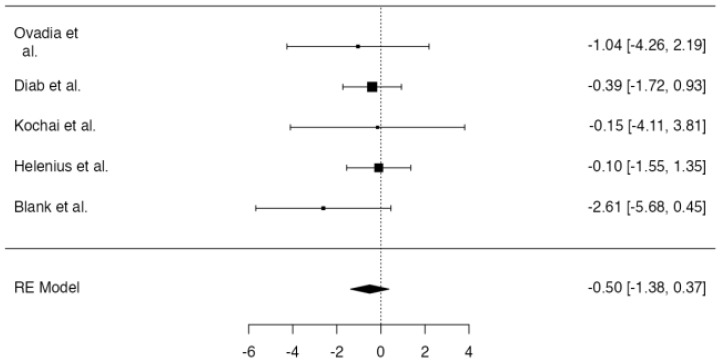
Forest plot of overall meta-analysis of the included studies with data about the need of reintervention [3,8,9,10,11].

**Figure 11:**
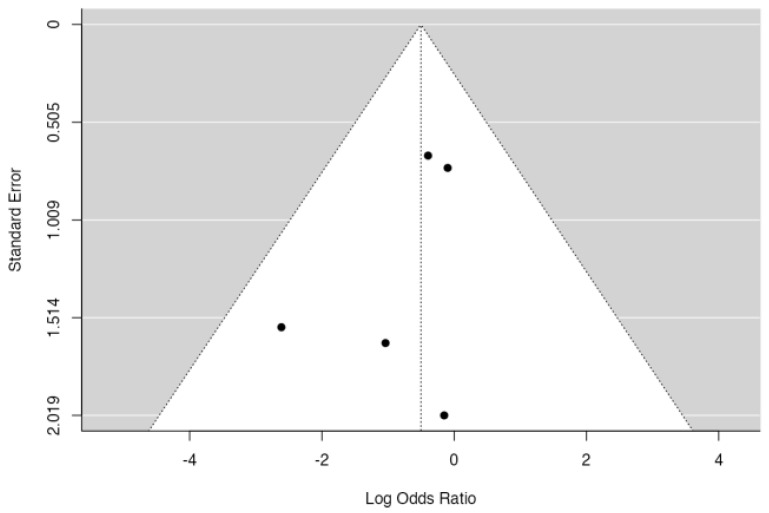
Funnel plot for assessing the presence of publication bias in the studies included in the meta-analysis regarding need of reintervention.

As for the need of reintervention due to any cause, patients in the no-drain group showed a tendency to higher reintervention rates, but overall, meta-analysis showed no significant difference in the reintervention rate (*p* = 0.260 Figure 10), with an estimated average log odds ratio of −0.503 (95% CI −1.377–0.371). No significative heterogeneity (I2 = 0.0, *p* = 0.684) or publication bias (*p* = 0.817, Figure 11) were found.

Details related to complication are highlighted in Table 3.

## 4. Discussion

The main finding of the present study is that the current available literature does not exhibit significant differences in short-term outcomes of AIS surgery whether the drain was applied or not, in terms of SSI, re-intervention rate, need for blood transfusion, and hospital length of stay.

Closed suction drainage in the setting of AIS surgery is intended to remove the blood and serum from the surgical site to avoid post-operative hematoma and subsequent consequences like wound complications, compression of exposed neural elements, and deep SSI [3,12]. To date, drains are mainly used based on the surgeon’s experience and intra-operative findings like quality of hemostasis achieved, invasiveness of the surgical procedure, and eventual exposure of the dura and neural elements. The advantages of drainage following AIS surgery are still debatable [4,13]. A consensus on whether using a drain or not in the setting of AIS surgery has not yet been reached.

Alongside the evolution of surgical techniques, post-operative protocols of AIS have also undergone major improvements. ERAS (enhanced recovery after surgery) protocols are nowadays applied in several institutions as multimodal and multidisciplinary approaches for improving perioperative outcomes of patients, reducing the hospital length of stay with an adjunctive positive financial impact on healthcare management. They imply multimodal analgesia, early patient mobilization, a bowel care regime, an early urinary catheter, and post-operative drain removal [14]. Intra-operative strategies of ERAS protocols often include avoidance of drains if possible [15]. Few authors reported no increase in blood loss, transfusion requirements, wound infection, skin dehiscence, and wound hematoma after AIS surgery without using drain [16]. Potential disadvantages of drains include impeded mobilization, increased discomfort, and increased need for post-operative care, such as stripping or reservoir emptying [3].

Previous literature has evaluated potential risks of drain use in lumbar spinal surgery and hip and knee arthroplasties, revealing a tendency for patients in which the drain was applied to receive more postoperative blood transfusions and to have a longer postoperative hospital stay [17,18,19,20,21].

Helenius et al., Diab et al., and Blank et al. reported higher overall transfusion rates in patients in the no-drain group [3,9,10], whereas Kochai et al. and Ovadia et al. reported a higher overall transfusion rate in patients from the drain group [8,11]. Overall meta-analysis showed a surprisingly slight tendency of patients in the no-drain group to require more blood transfusions overall. Some authors hypothesize that drainage prevents tamponade, resulting in greater post-operative blood loss and a subsequent higher blood transfusion rate [9]. Although closed suction drainage may obstruct tamponade following hip or knee arthroplasty, this phenomenon might be of weaker importance in patients undergoing AIS surgery, primarily because of prolonged post-operative supine positioning that simultaneously results in compression of the surgical site, reducing dead space, and thus mechanically tamponing the bleeding. This fact becomes more relevant when the spinous processes and posterior elements are left intact, allowing for an effective bony attachment of paravertebral muscle. Helenius et al. [10] reported that around 15% of patients required posterior column osteotomies (PCOs) that are known to increase post-operative blood loss and requirement for blood transfusion [22], and Diab et al. [3] reported that around 10% of patients underwent thoracoplasties. In addition, overall transfusion rates may be influenced by many variables, firstly, the intra-operative transfusion rate. Evaluation of the post-operative transfusion rate alone was not possible due to lack of data in the included studies. The overall transfusion rate was regarded as an estimate of the post-operative transfusion rate, as there was no statistically significant difference between the drain and no-drain group in terms of intraoperative transfusion rates in any of the included studies. No difference in overall transfusion rates was found at meta-analysis between patients in the drain and no-drain groups. However, the limitations in the included studies make this data difficult to explain, as the comparability of patient groups is hindered by the lack of data regarding the invasiveness of the applied procedures, despite the comparability in the severity of the deformity and the average number of fused levels for all the studies, as shown in Table 2.

As for hospital length of stay, overall meta-analysis showed a moderately longer length of stay in patients of the drain group, with a standardized mean difference of +0.22 days. Ovadia et al. [8] reported longer LOS in patients of no-drain group. However, statistical significance was not reached; this finding is still important since in the current literature, the average difference in LOS between traditional postoperative protocols and ERAS protocols was 1.4–1.6 days. No ERAS protocol was reported to be applied in the included studies. Viewed in this perspective, the data may indicate that the absence of drainage might account for up to a 15% reduction in LOS and the significance of the data might not become evident in a setting that does not implement fast track protocols.

As for complications, Helenius et al., Ovadia et al., and Diab et al. reported a higher SSI rate among patients in the no-drain group, whereas Kochai et al. reported a higher SSI rate in patients from the drain group [3,8,10,11]. Overall meta-analysis showed a slight tendency for patients in whom a drain was not applied to be more likely to develop SSI, although statistical significance was not reached. The higher surgical site infection rate in one group compared to the other can be explained by two opposite pathophysiological mechanisms. The first involves the drain, which acts as a potential pathway for external contamination to reach the surgical site. The second involves hematoma formation that can facilitate bacterial growth due to a relative hypoxic environment. Looking at the need for re-intervention, Helenius et al., Diab et al., and Blank et al. reported a higher re-intervention rate in the no-drain group [3,9,10], whereas Kochai et al. [11] reported higher need for re-intervention in the drain group. Overall meta-analysis showed a slight inclination of patients in the no-drain group to require re-intervention with a higher probability. Nevertheless, statistical significance was not reached. Notably, the need for surgical revision involves numerous variables and may be attributed to various factors or complications, not limited solely to wound-related issues. Further risks of drainage could be associated with its misplacement, causing pain or compression of neural elements [23].

The studies included in the present work did not show significant differences in overall transfusion rate, SSI rate, length of hospital stay, or overall re-intervention rate at meta-analysis. Consequently, no notable differences were observed in the assessed short-term outcomes of AIS surgery based on whether a drain was used or not. The present study confirms that drain placement should be based on personal experience and intraoperative findings. Furthermore, the drain placement is of mainstay importance in cases involving accessory procedures such as osteotomies and exposure of the dural sac and neural elements.

The present study does not come without limitations. Firstly, the complications resulting from hematoma formation, which is believed to be the main non-infectious complication risk when drainage is not used, could not be evaluated due to the lack of data in the included studies.

The comparability of patients and surgical techniques was limited. Th eincluded studies are heterogeneous regarding study design, randomization of patients, type of spinal instrumentation, accessory procedures applied, drain type (subfascial and subcutaneous), drain placement criteria, drain removal criteria, and surgical technique. Evaluation of post-operative estimated blood loss and post-operative transfusion rate was not possible due to lack of data in the included studies. the included studies are heterogeneous in the formulae used for estimated intra-operative blood loss. In addition, the included studies showed relative inconsistency in the reported outcomes between the drain and no-drain groups that may arise from the variations in study methodologies and patient populations.

The variability in the above-mentioned factors may limit the generalizability of findings.

Finally, the present meta-analysis includes both retrospective comparative studies and randomized clinical trials, which may possibly represent an additional source of bias.

Despite that, to the best of our knowledge, this is the first meta-analysis regarding drainage use in adolescent idiopathic scoliosis surgery.

Further clinical trials that implement the ERAS protocol are required to better evaluate the difference in post-operative outcomes between patients with drains and those without.

## 5. Conclusions

The present systematic review and meta-analysis found no significant differences in short-term outcomes between adolescent idiopathic scoliosis (AIS) patients treated surgically with or without drainage. This suggests that the choice of drainage should rely on personal experience and intra-operative findings. However, the study has limitations and underscores the need for further research, particularly with ERAS or fast track protocols, to better understand the role of drainage in AIS surgery.

## Figures and Tables

**Table 1:** Summary of selected studies with characteristics of patients’ clinical outcomes of interest. ✓: yes; ✕: no.

Author		Study Design	Level of Evidence	Patients N° (M/F)		Inclusion Criteria	Mean Patients Age (Years)	Closed Suction Drain Type	Autologous Bone Harvest (Eventual Donor Site Drain Placement)	Intraoperative Cell Saver	Intrawound Antibiotics	Perioperative Antibiotics Protocol	Mean Surgical Time (min)	Mean Intraoperative Blood Loss (mL)	Mean Post-Operative Blood Loss (mL)/Drain Volume at Removal	Drain Removal Criteria/Drain Removal Time	Transfusion Criteria	Intra-Operative Transfusion	Post-Operative Transfusion	Overall Transfusion	Mean Length of Stay (Days)	Overall Complication (*n*, %)	Surgical Site Infection (*n*, %)	Hematoma (*n*, %)	Overall Revision Surgery Needed (*n*, %)	Main Findings
**1**	Helenius, 2022 [10] (D)	Multi-center RCT	II	90 (22/68)	47 (12/35)	AIS patients of age 10–21. Patients who required ncreas Schwab > 2 osteotomies or combined approaches were excluded	15.7 ± 2.0	Subfascial closed suction Hemovac Ch 14 drain (Zimmer, Dover, OH, USA)	/	/	/	Cefuroxime or cloxacilline were continued for 3 doses post-operatively	180 ± 60	565.0 ± 339.0	443.0 ± 520.0	24 h post-operatively	Hb < 8 g/dL	2	1	3	4.8 ± 1.3	2 (4.3%)	1 (2.1%)	/	0	Subfascial closed suction drainage increased total blood loss but did not affect postoperative haemoglobin drop or need for blood transfusion. Patients in the non-drain group needed 30% more opioid during the first 48 h after surgery. A significant subfascial haematoma develops in patients who do not receive drainage. However, the drain itself does not increase postoperative bleeding.
	Helenius, 2022 [10] (ND)				43 (10/33)		15.8 ± 1.8	/		/	/		186 ± 60	603.0 ± 423.0	/	/		1	4	5	4.7 ± 1.7	3 (7.0%)	2 (4.6%)	/	1 (2.3%)	
**2**	Kochai, 2019 [11] (D)	RCS	III	52 (20/32)	28 (13/15)	AIS patients of age 14–18. Patients who required Schwab > 2 osteotomies and post-operative intensive care admission were excluded.	15 (14–16)	Subfascial closed suction 2–4 mm drain tube	/	/	/	48 h course of antibiotic post-operatively	/	/	400.0 (350.0–500.0)	Output < 50 mL/die 36 h (30–40)	Hb < 8.5 g/dL	2		2	6 (6–7)	5 (17.8%)	5 (17.8%)	0	5 (17.8%)	No significant difference in infection rate between patients who received closed subfascial drains and patients without any drainage. Patients in the drain group showed significantly longer hospital stay and higher blood transfusion rate.
	Kochai, 2019 [11] (ND)				24 (7/17)		14.5 (14–16)	/		/	/		/	/	/	/		0		0	5 (5–5)	4 (16.6%)	4 (16.6%)	0	4 (16.6%)	
**3**	Ovadia, 2019 [8] (D)	RCT	II	100 (22/78)	48 (13/35)	AIS patients of age 11–18, with the major curve Cobb angle of more than 50°, and the absence of intradural abnormalities at whole spine MRI	15.8 ± 1.9	Subfascial closed suction drain (Biovac Biometrix, Gronsveld, The Netherlands)	✕	✓	1 g of vancomycin powder applied into the wound	/	207.9 ± 55.1	/	974.0 ± 248.6	Output < 100 mL/die, as of the second or third postoperative day 56.9 ± 13.7 h	/	3		3	5.81 ± 0.7	1 (2.1%)	0	0	0	No significant differences in short-term complication rates between patients who received closed subfascial drains and patients without any drainage. Drains did not significantly reduce wound infection and wound dehiscence rate. Patients who received drainage were more likely to have larger post-operative bleeding and, subsequently, blood transfusions.
	Ovadia, 2019 [8] (ND)				52 (9/43)		15.3 ± 2.0	/					205.0 ± 45.0	/	/	/		1		1	6.1 ± 1.3	4 (7.7%)	2 (3.8%)	0	0	
**4**	Diab, 2012 [3] (D)	Multi-center RCS	III	500 (105/395)	324 (62/262)	AIS patients of age 13–21 years at surgery with Risser sign > 2. Minimum 2 years follow up	15.7 ± 1.6	Subfascial closed suction drainage: 22 Subcutaneous closed suction drainage: 176 Combined deep and superficial drainage: 107	/	✓: 193 ✕: 131	/	/	275.8 ± 80.6	803.2 ± 598.1	775.5 ± 581.2	57.2 ± 25.8 h after surgery	/	237	131	267	5.9	18 (5.5%)	6 (1.8%)	/	4 (1.2%)	Wound drains were used twice as often as not by a heterogeneous group of spinal surgeons. Patients with all-pedicle screw construct were more likely to receive drains. Postoperative transfusion rate and mean amount of blood transfused were higher in patients who had drain. Postoperative transfusion rate correlated with number of drains. There was no difference in incidence of wound complications.
	Diab, 2012 [3] (ND)				176 (43/133)		15.6 ± 1.7	/		✓: 129 ✕: 47			306.9 ± 78.1	900.4 ± 719.4	/	/		150	38	156		19 (10.8%)	5 (2.8%)	/	4 (2.3%)	
**5**	Blank, 2003 [9] (D)	RCT	II	30 (4/26)	18	Consecutive AIS patients who underwent PSF	14.4 (11–17)	Subcutaneous Hemovac drain (Zimmer, Dover, OH, USA)	Iliac crest autogenous bone harvest (closed suction subfascial drain at the iliac donor site, separate reservoir)	✓	✕	48 h of cephalosporin beginning intraoperatively	/	887.5 ± 356.7	548.4	48 h after surgery	Hb < 8 g/dL or if patients exhibit anemia signs and symptoms	14	11	17	/	0	0	0	0	Closed suction drainage can decrease wound complications, without significantly increasing the need for transfusion. Furthermore, the use of drainage may reduce the frequency of required dressing change or reinforcement. Subcutaneous drainage did not significantly increase blood loss.
	Blank, 2003 [9] (ND)				12		13.3 (11–16)	/						1091.6 ± 457.2)		/		11	5	12		3 (25%)	/	/	3 (25%)	

**Table 2:** Details of surgical procedures of the studies analyzed in this meta-analysis.

Authors	Drainage	Lenke Types	Mean Pre-Operative Cobb Angle of Major Curve (°)	Mean Post-Operative Cobb Angle of Major Curve (°)	Internal Fixation System		Osteotomies/Accessory Procedures	Mean Fused Levels (*n*)
Helenius, 2022 [10]	D	I: 17 II: 13 III: 4 IV: 1 V: 8 VI: 4	55.0 ± 8.4	15.0 ± 7.0	All-pedicle screw		7 PCOs (15%)	10.4 ± 2.4
	ND	I: 16 II: 13 III: 0 IV: 7 V: 3 VI: 4	56.0 ± 8.2	17.0 ± 6.0			7 PCOs (16%)	10.8 ± 1.9
Kochai, 2019 [11]	D	I: 12 II: 10 III: 1 V: 5	/	/	/		/	11 (10–12)
	ND	I: 13 II: 5 III: 4 V: 2						11 (11–12)
Ovadia, 2019 [8]	D	I: 29 II: 4 III: 6 IV: 0 V: 3 VI: 6	64.7 ± 12.5	/	All-pedicle screw		No osteotomies. The spinous processes were left intact. No iliac crest harvesting.	/
	ND	I: 24 II: 4 III: 10 IV: 2 V: 7 VI: 5	65.4 ± 15.0					
Diab, 2012 [3]	D	I: 153 II: 66 III: 35 IV: 12 V: 34 VI: 24	56.7 ± 12.1	/	All-pedicle screw: 182 Hybrid construct: 291 All-hook: 23	All-pedicle screw: 164 Hybrid construct: 153 All-hook: 7	10% of thoracoplasties. No complex osteotomies or VCR.	11.7 ± 2.3
	ND	I: 91 II: 50 III: 10 IV: 4 V: 11 VI: 10	56.9 ± 10.6			All-pedicle screw: 18 Hybrid construct: 138 All-hook: 16	6% of thoracoplasties. No complex osteotomies or VCR.	11.2 ± 2.4
Blank, 2003 [9]	D	/	/	/	Two-rod and cross-linked construct		/	7.6 (5–10)
	ND							8.8 (7–11)

**Table 3:** Detailed characteristics of perioperative complications in the studies analyzed in this meta-analysis.

Authors	Drainage	No. of Patients	Complication Overall (*n*)	Surgical Site Infection (*n*)	Wound-Related Complications (*n*)	Medical Complication (*n*)	Mechanical Complication (*n*)	Neurological Complication (*n*)
Helenius, 2022 [10]	D	47	2 (4.3%)	1 (2.1%) Superficial SSI positive for *Staphylococcus aureus*	/	/	/	/
	ND	43	3 (7.0%)	2 (4.6%): 1 deep SSI 1 superificial SSI both positives for *Staphylococcus aureus*	1 (2.3%) sterile seroma which required aspiration 13 days after surgery	/	/	/
Kochai, 2019 [11]	D	28	5 (17.8%) Negative coltures	5 (17.8%) superificial SSI	/	/	/	/
	ND	24	4 (16.6%) Negative coltures	4 (16.6%) superificial SSI	/	/	/	/
Ovadia, 2019 [8]	D	48	1 (2.1%)	0	1 (2.1%) wound dehiscence	/	/	/
	ND	52	4 (7.7%)	2 (3.8%) superficial SSI	1 (1.9%) wound dehiscence	1 (1.9%) pneumonia	/	/
Diab, 2012 [3]	D	324	18 (5.5%)	6 (1.8%)	/	6 (1.8%) 1 urinary tract infection 4 re-operation 1 superior mesenteric artery syndrome	2 (0.6%) 1 implant failure 1 pedicle fracture requiring re-intervention	4 (1.2%) 2 nerve root injury 1 spinal cord injury 1 CSF leak
	ND	176	19 (10.8%)	5 (2.8%)	/	7 (4.0%) 1 pneumonia 3 re-operation 1 superior mesenteric artery syndrome 1 respiratory distress syndrome 2 other	2 (1.1%) implant failure	5 (2.8%) 1 nerve root injury 3 radiculopathy 1 CSF leak
Blank, 2003 [9]	D	18	0	0	/	/	/	/
	ND	12	3 (25%)	/	3 (25%) wound complication	/	/	/

## Data Availability

The data presented in this study are available on request from the corresponding author.

## References

[1] Lenke L.G., Betz R.R., Harms J., Bridwell K.H., Clements D.H., Lowe T.G., Blanke K. (2001). Adolescent Idiopathic Scoliosis. A New Classification to Determine Extent of Spinal Arthrodesis. J. Bone Jt. Surg..

[2] Bettany-Saltikov J., Weiss H.R., Chockalingam N., Taranu R., Srinivas S., Hogg J., Whittaker V., Kalyan R.V., Arnell T. (2015). Surgical versus Non-Surgical Interventions in People with Adolescent Idiopathic Scoliosis. Cochrane Database Syst. Rev..

[3] Diab M., Smucny M., Dormans J.P., Erickson M.A., Ibrahim K., Lenke L.G., Sucato D.J., Sanders J.O. (2012). Use and Outcomes of Wound Drain in Spinal Fusion for Adolescent Idiopathic Scoliosis. Spine.

[4] Parker M.J., Livingstone V., Clifton R., McKee A. (2007). Closed Suction Surgical Wound Drainage after Orthopaedic Surgery. Cochrane Database Syst. Rev..

[5] Page M.J., McKenzie J.E., Bossuyt P.M., Boutron I., Hoffmann T.C., Mulrow C.D., Shamseer L., Tetzlaff J.M., Akl E.A., Brennan S.E. (2021). The PRISMA 2020 Statement: An Updated Guideline for Reporting Systematic Reviews. BMJ.

[6] Sterne J.A., Hernán M.A., Reeves B.C., Savović J., Berkman N.D., Viswanathan M., Henry D., Altman D.G., Ansari M.T., Boutron I. (2016). ROBINS-I: A Tool for Assessing Risk of Bias in Non-Randomised Studies of Interventions. BMJ.

[7] Sterne J.A.C., Savović J., Page M.J., Elbers R.G., Blencowe N.S., Boutron I., Cates C.J., Cheng H.Y., Corbett M.S., Eldridge S.M. (2019). RoB 2: A Revised Tool for Assessing Risk of Bias in Randomised Trials. BMJ.

[8] Ovadia D., Drexler M., Kramer M., Herman A., Lebel D.E. (2019). Closed Wound Subfascial Suction Drainage in Posterior Fusion Surgery for Adolescent Idiopathic Scoliosis: A Prospective Randomized Control Study. Spine.

[9] Blank J., Flynn J.M., Bronson W., Ellman P., Pill S.G., Lou J.E., Dormans J.P., Drummond D.S., Ecker M.L. (2003). The Use of Postoperative Subcutaneous Closed Suction Drainage after Posterior Spinal Fusion in Adolescents with Idiopathic Scoliosis. J. Spinal Disord. Tech..

[10] Helenius L., Gerdhem P., Ahonen M., Syvänen J., Jalkanen J., Charalampidis A., Nietosvaara Y., Helenius I. (2022). Postoperative Outcomes of Pedicle Screw Instrumentation for Adolescent Idiopathic Scoliosis with and without a Subfascial Wound Drain: A Multicentre Randomized Controlled Trial. Bone Jt. J..

[11] Kochai A., Erkorkmaz Ü. (2019). The Role of Drains in Adolescent Idiopathic Scoliosis Surgery: Is It Necessary?. Medicine.

[12] Andrew Glennie R., Dea N., Street J.T. (2015). Dressings and Drains in Posterior Spine Surgery and Their Effect on Wound Complications. J. Clin. Neurosci..

[13] Patel S.B., Griffiths-Jones W., Jones C.S., Samartzis D., Clarke A.J., Khan S., Stokes O.M. (2017). The Current State of the Evidence for the Use of Drains in Spinal Surgery: Systematic Review. Eur. Spine J..

[14] Gadiya A.D., Koch J.E.J., Patel M.S., Shafafy M., Grevitt M.P., Quraishi N.A. (2021). Enhanced Recovery after Surgery (ERAS) in Adolescent Idiopathic Scoliosis (AIS): A Meta-Analysis and Systematic Review. Spine Deform..

[15] Koucheki R., Koyle M., Ibrahim G.M., Nallet J., Lebel D.E. (2021). Comparison of Interventions and Outcomes of Enhanced Recovery after Surgery: A Systematic Review and Meta-Analysis of 2456 Adolescent Idiopathic Scoliosis Cases. Eur. Spine J..

[16] Hassankhani E.G., Hassankhani G.G., Pharmsist S.G.H. (2023). The Outcome of Posterior Spinal Fusion and Instrumentation of Adolescent Idiopathic Scoliosis without Wound Suction Drainage. Open J. Orthop..

[17] Zhang Q.D., Guo W.S., Zhang Q., Liu Z.H., Cheng L.M., Li Z.R. (2011). Comparison between Closed Suction Drainage and Nondrainage in Total Knee Arthroplasty. A Meta-Analysis. J. Arthroplast..

[18] Albasha A., Salman L.A., Elramadi A., Abudalou A., Mustafa A., Hejleh H.A.A., Ahmed G. (2023). Outcomes of Drain versus No Drain in Total Knee Arthroplasty: A Retrospective Cohort Study. Int. Orthop..

[19] Zhou X.D., Li J., Xiong Y., Jiang L.F., Li W.J., Wu L.D. (2013). Do We Really Need Closed-Suction Drainage in Total Hip Arthroplasty? A Meta-Analysis. Int. Orthop..

[20] Fichman S.G., Mäkinen T.J., Lozano B., Rahman W.A., Safir O., Gross A.E., Backstein D., Kuzyk P.R.T. (2016). Closed Suction Drainage Has No Benefits in Revision Total Hip Arthroplasty: A Randomized Controlled Trial. Int. Orthop..

[21] Walid M.S., Abbara M., Tolaymat A., Davis J.R., Waits K.D., Robinson J.S., Robinson J.S. (2012). The Role of Drains in Lumbar Spine Fusion. World Neurosurg..

[22] Floccari L.V., Poppino K., Greenhill D.A., Sucato D.J. (2021). Ponte Osteotomies in a Matched Series of Large AIS Curves Increase Surgical Risk without Improving Outcomes. Spine Deform..

[23] Barile F., Ruffilli A., Viroli G., Manzetti M., Traversari M., Faldini C. (2022). Transient L5 Nerve Root Palsy Caused by Subfascial Drain after Lumbar Surgery: Case Report and Literature Review. JBJS Case Connect.

